# PIAS1 is not suitable as a urothelial carcinoma biomarker protein and pharmacological target

**DOI:** 10.1371/journal.pone.0224085

**Published:** 2019-10-22

**Authors:** Holger Hans Hermann Erb, Marlies Ebert, Ronja Kuhn, Lukas Donix, Axel Haferkamp, Robert Ian Seed, Eva Jüngel

**Affiliations:** 1 Department of Urology and Pediatric Urology, University Medical Center Mainz, Mainz, Germany; 2 Department of Urology, Technische Universität Dresden, Dresden, Germany; 3 National Center for Tumor Diseases (NCT), Partner Site Dresden, Germany: German Cancer Research Center (DKFZ), Heidelberg, Germany; Faculty of Medicine and University Hospital Carl Gustav Carus, Technische Universität Dresden, Dresden, Germany and Helmholtz Association/Helmholtz-Zentrum Dresden - Rossendorf (HZDR), Dresden, Germany; 4 Department of Pathology, University of California, San Francisco, California, United States of America; Medizinische Universitat Innsbruck, AUSTRIA

## Abstract

Urothelial cancer (UC) is one of the most common cancers in Europe and is also one of the costliest to treat. When first line therapies show initial success, around 50% of cancers relapse and proceed to metastasis. In this study we assessed the Protein inhibitor of activated signal transducers and activators of transcription (PIAS)1 as a potential therapeutic target in urothelial cancer. PIAS1 is a key regulator of STAT1 signalling and may be implicated in carcinogenesis. In contrast to other cancer types PIAS1 protein expression is not significantly different in malignant areas of UC specimens compared to non-malignant tissue. In addition, we found that down-regulation and overexpression of PIAS1 had no effect on the viability or colony forming ability of tested cell lines. Whilst other studies of PIAS1 suggest an important biological role in cancer, this study shows that PIAS1 has no influence on reducing the cytotoxic effects of Cisplatin or cell recovery after DNA damage induced by irradiation. Taken together, these *in vitro* data demonstrate that PIAS1 is not a promising therapeutic target in UC cancer as previously shown in different entities such as prostate cancer (PCa).

## Introduction

Europe has one of the highest incidence rates of bladder cancer (BC) in the world, the majority of which are urothelial cancer (UC) [1]. Current gold-standard treatment for UC is the surgical removal of the bladder (radical cystectomy). However, ~50% patients will still relapse and proceed to develop metastasis [2]. Currently, patients with metastatic UC receive platinum-based cisplatin chemotherapy and/or radiotherapy (RT) as non-invasive therapy options either before or after cystectomy [3]. However, the success of these non-invasive therapies is still sub-optimal, and more efficient treatment protocols need to be developed.

DNA repair mechanisms play an important role in the response of cancer cells to RT or cisplatin treatment, and in the development of therapy resistance [4]. These mechanisms can remove the bulky, helix-distorting DNA adducts induced by cisplatin, as well as the DNA breaks caused by ionizing radiation [5]. Protein Inhibitor of Activated STAT (PIAS)1 has been shown to play an important role in the repair of cisplatin-induced DNA cross-links and radiation-induced DNA strand breaks [6, 7]. PIAS1 belongs to the multifunctional PIAS protein family that play a role in the regulation of cytokines and other cellular pathways [8]. Besides its ability for DNA and protein binding via its conserved SAP domain, PIAS1 also contains a RING finger-like zinc binding domain (RLD) and a SUMO interaction motif (SIM), thus functioning as a SUMO-E3 ligase [8]. Therefore, PIAS1 can influence the activity of various proteins and signalling cascades. In breast and prostate cancer PIAS1 has been reported to be involved in cancer progression and appears to be a valid target for cancer therapy even in resistant cells [9–12]. However, there are currently no studies investigating either the role of PIAS1 in UC or in the development of treatment resistance.

The aim of this study is to investigate the potential role of PIAS1 in UC for the first time, and whether it may function to regulate the urothelial cell DNA damage response induced by therapeutic approaches.

## Materials and methods

### Data mining

For mutation analysis of PIAS1 the The Cancer Genome Atlas (TCGA, https://portal.gdc.cancer.gov/) data base was used. For PIAS1 expression analysis the dataset GSE27448, GSE3167, and GSE13507 were analysed by using GEO2R (https://www.ncbi.nlm.nih.gov/geo/geo2r/) [13–15].

### Cell lines and cell culture

UROtsa, RT112, TCCSUP, T24, Cal-29 and RT4 cells were obtained from the ATCC. All cells with exception of Cal-29 were cultured in RPMI 1640 medium (Seromed, Berlin, Germany) supplemented with 10% fetal calf serum (FCS), 20 mM HEPES-buffer, 1% glutamax, and 1% penicillin/streptomycin (all: Gibco/Invitrogen; Karlsruhe, Germany). Cal-29 were cultured in Dulbecco’s Modified Eagle’s Medium (Sigma-Aldrich, Taufkirchen, Deutschland) supplemented with 10% FCS, 20 mM HEPES-buffer, 1% glutamax, and 1% penicillin/streptomycin (all: Gibco/Invitrogen; Karlsruhe, Germany).

### Live cell count

Collected cells were stained with Trypan Blue (Sigma-Aldrich Chemie GmbH; Munich; Germany) and counted using a LuncaTM Second Generation Automated Cell Counter (logos Biosystems, Villeneuve d’Ascq, Frankreich).

### RNA isolation and quantitative real-time PCR

Quantitative real-time PCR (qRT-PCR) experiments were performed in six-well plates. Cells were seeded in a density of 500,000 cells/well and were harvested after 48 h. RNA was isolated using the RNeasy Plus Mini Kit by following the manufacturer’s instructions (Qiagen). cDNA synthesis was performed using iScript select cDNA synthesis kit (Bio-Rad). qRT-PCR was performed using the MIC qPCR cycler (Bio Molecular Systems, Upper Coomera, Australia) and TaqMan gene expression assays for PIAS1 and HRPT1 (both Applied Biosystems). HPRT1 was used as a control. micPCR software was used for determination of Ct values. ΔCt (ΔCt = (*CtGOI* − *CtHRPT*1)) values were calculated and expressed as 2^-ΔCt^.

### Irradiation of cells

Cells were irradiated using a Gammacell 2000 (Nuklear Data, Frankfurt, Germany) at the Medical University of Mainz (Dr. Jürgen Podlech, Institute of Virology). A dose of 2.5, 5, 10, 20 or 40 Gy was administered with a dose rate of 0.044 Gy/s.

### Western blot analysis

For western blot analysis cells were washed with Phosphate-buffered saline (PBS) and lysed in Radioimmunoprecipitation assay (RIPA) buffer with complete Mini EDTA-free protease inhibitor tablets (Roche, Welwyn Garden City, UK) and the phosphatase inhibitor cocktail PhosSTOP (Roche, Welwyn Garden City, UK). The protein quantification and western blot was performed as described earlier [16]. PIAS1 XP^®^ Rabbit mAb (1:500, D33A7, Cell Signaling, Frankfurt am Main, Germany), Anti-β-Actin monoclonal (1:5000, AC-15, Sigma-Aldrich GmbH; Munich; Germany) and Anti-Lamin A antibody (1:1000, Abcam, Cambridge, UK) were used as primary antibody. Rabbit Anti-Mouse IgG, HRP and Goat anti-rabbit IgG, HRP (both: 1:1000, Dako, Frankfurt, Germany) were used as secondary antibody.

### Cytoplasmatic and nuclear fractionation

Fractions were obtained using NE-PER nuclear and cytoplasmic extraction kit (Pierce, Vienna, Austria) following the manufacturer’s instructions.

### Immunofluorescence

Cells were seeded onto glass coverslips and allowed to attach for 24 h. Depending on the assay, they were grown for 2 d without treatment (for localization studies) or transfected with siRNA against PIAS1 or control siRNA (for siRNA efficiency studies and p21 expression experiments). Subsequently, the cells were again washed with PBS and fixed with 4% paraformaldehyde for 10 min. The cells were washed with PBS and permeabilized with PBS+1% bovine serum albumin+0.2% Triton X100 for 5 min. After 30 min blocking with PBS+1% bovine serum albumin, coverslips were incubated for 1 h with primary antibodies (PIAS1 [D33A7] XP^®^ Rabbit mAb 1:50, Cell Signaling, Frankfurt am Main, Germany). After washing with TBS + 0.1% Tween20, coverslips were incubated with the following fluorescence-labeled secondary antibodies: goat anti-rabbit 555 and donkey anti-mouse 488 (all Invitrogen). Coverslips were finally washed with TBS and mounted with Vectashield Hard Set mounting medium containing DAPI (Vector Laboratories, Burlingame, CA) on glass slides. The cells were visualized using fluorescent microscopy on an Axio Observer Z1 microscope (Carls Zeiss AG, Overkochen, Germany).

### Transfections

For siRNA transfections, 50 nmol/l FlexiTube GeneSolution GS8554 for PIAS1 (QIAGEN GmbH, Hilden, Deutschland) were used (PIAS1_6: SI02641975; PIAS1_7: SI02641968). The AllStars Negative Control siRNA served as control (QIAGEN GmbH, Hilden, Deutschland). Transfections of siRNA were performed with Lipofectamine RNAiMax (Invitrogen) according to the manufacturer’s protocol. For overexpression experiments, the plasmid pEGFP-C1-PIAS1wt (generated by Dr. Yaron Galanty; Gurdon Institute, Cambridge, UK) and empty vector pEGFP-C1-CTRL were used. Depending on the well size, the cells were transfected with 1 μg/ml plasmid in 6-well plates and 0.1 μg/ml in 96-well plates using ViaFect^™^ Transfection Reagent transfection reagent (Promega GmbH High-Tech-Park, Mannheim, Deutschland) for 48 or 72 h following the manufacturer’s instructions.

### Measurement of cell viability

Resazurin sodium salt (Sigma-Aldrich Chemie GmbH; Munich; Germany) was used to carry out alamar blue assays. A 25 mmol/L stock was diluted 50‐fold to generate a 10-fold working stock. Cells were plated at the stated number (5×10^3^) per well for cell line in 96‐well plates and incubated with drug. 4 h before mesurment, one‐tenth volume of the 10-fold working stock was added to each well and incubated for 4 h. Fluorescence (560EX nm/590EM nm) and absorbance (570 nm) was measured using a SPARK 10M Microplate reader (TECAN, Männedorf, Switzerland). Each experiment was done in triplicate. After subtracting background absorbance, results were expressed as cell growth occurring from 24 to 72 h, calculated in percentage increase from controls set to 100%.

### Clonogenic and clonogenic recovery assays

The clonogenic potential and clonogenic recovery assays were performed and analysed as described previously [17]. 100 cells/well were given in 6-well plates. Colonies were counted after 10 d and recorded if they contained more than 32 cells (five population doublings).

### Wound heal assay

2x10^4^ cells per well were seeded onto IncuCyte^®^ ImageLock 96-well plates (Essense Bioscience, Hertfordshire, United Kingdom) and incubated overnight. Subsequently cells were transfection with siRNA. Experiments were seeded as four technical replicates for each condigion. 24 h after transfection the IncuCyte^®^ 96-well WoundMaker Tool (Essense Bioscience, Hertfordshire, United Kingdom) was used to make a scratch. Wound closure was observed using the IncuCyte^®^ S3 Live-Cell Analysis System Tool (Essense Bioscience, Hertfordshire, United Kingdom). Wound closure was observed for 24 h. Pictures at 10-fold magnification were taken every 2 h. Wound closure was analysed using the Cell Migration Analysis software module (Essense Bioscience, Hertfordshire, United Kingdom).

### Drug treatment

Cisplatin (Sigma-Aldrich Chemie GmbH; Munich; Germany) was dissolved in DMSO as a 10 mM stock solution and stored as aliquots at– 20 °C. Prior to experiments, Cisplatin was diluted in cell culture medium. Cell growth experiments were carried out in the presence of 0, 2, 6, 12.5, 25 μg/ml Cisplatin for T24 and 0, 7.5, 12.5, 25 μg/ml Cisplatin for RT112. Controls remained untreated.

### Statistical analysis

Prism 8.02 (GraphPad Software, La Jolla, CA, USA) was used for statistical analyses. Student’s t-test (two-sided), one way Anova, or two-way ANOVA were used to determine whether two sets of data were significantly different from each other. Data are presented as mean±SD or mean±SEM unless otherwise specified. P-values ≤0.05 were considered significant. All differences highlighted by asterisks were statistically significant as encoded in figure legends (*P≤0.05; **P≤0.01; ***P≤0.001). All experiments were performed in at least three independent biological replicates.

## Results

### PIAS1 expression is not altered between benign and malignant urothelial tissue

**Fig 1:**
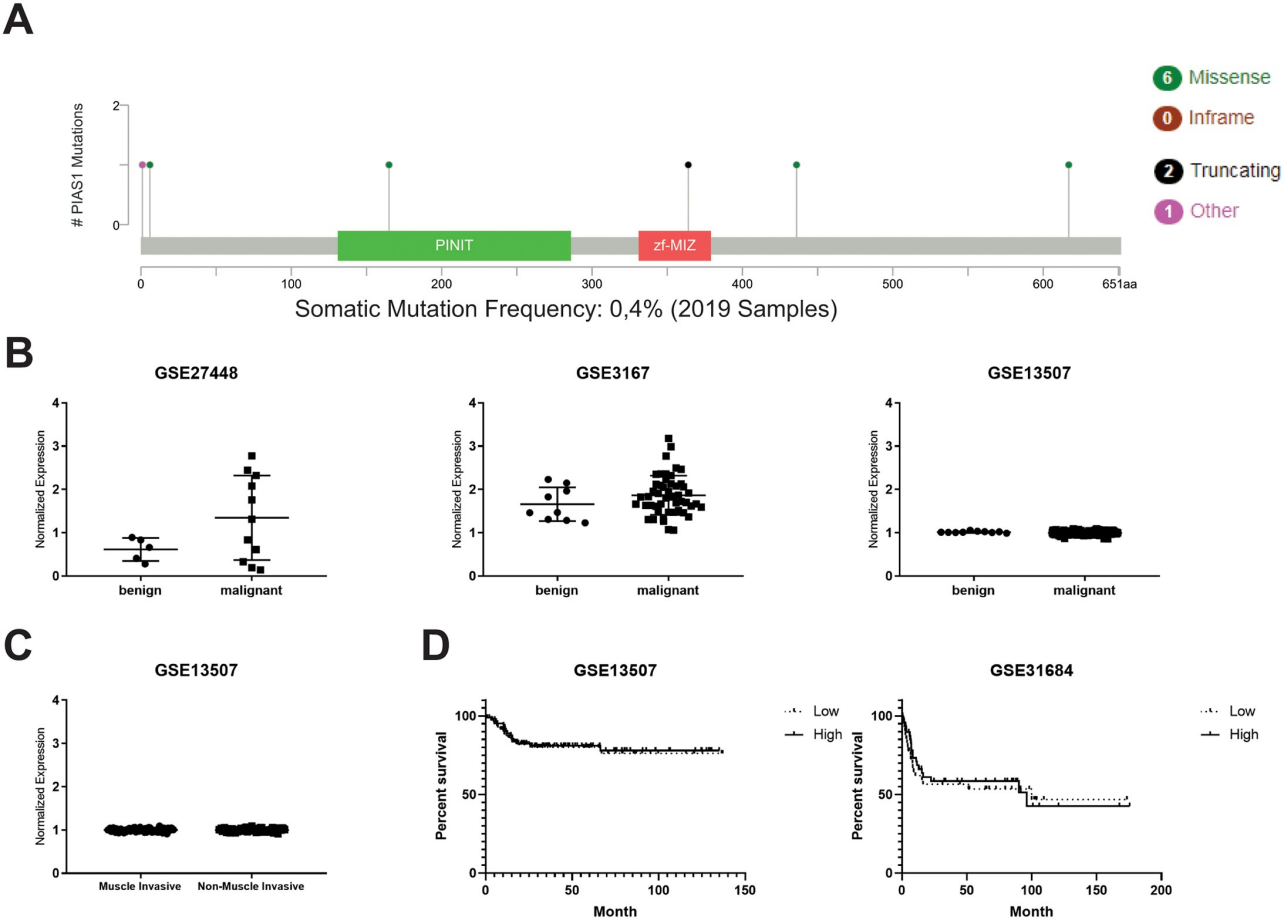
PIAS1 expression is not altered between benign and malignant urothelial tissue. (A) Mutation analysis of PIAS1 in benign and malignant urothelial tissue (B) mRNA expression profiles of PIAS1 in benign and malignant areas from the GSE27448, GSE3167 and GSE13507 data sets. (C) mRNA expression profiles in muscle invasive and non-muscle invasive UC from the GSE13507 data set. (D) Recurrence free survival analysis from publically available datasets (GSE13507, GSE31684) patients with low and high PIAS1 levels. The median expression level was chosen as threshold.

To investigate PIAS1 in benign and malignant human urothelial tissue, mutation rate and expression was analysed. Mutation analysis (Fig 1A) of 2019 patients revealed a somatic mutation rate of 0.4% [18, 19]. To analyse PIAS1 expression in benign and malignant urothelial tissue, three different already published microarrays (GSE27448, GSE3167, GSE13507) were analysed [13–15]. As shown in Fig 1B, PIAS1 mRNA is expressed heterogeneously in malignant areas. However, there was no significant difference between benign and malignant human urothelial tissue (Fig 1B). PIAS1 also showed no difference in mRNA expression when muscle invasive and non-muscle invasive samples where compared (Fig 1C). Kaplan-Meier survival analysis on publically available datasets (GSE13507, GSE31684) also revealed that expression of PIAS1 had no influence on recurrence free survival (Fig 1D).

### PIAS1 is expressed in the nucleus of benign and malignant urothelial cell lines

**Fig 2:**
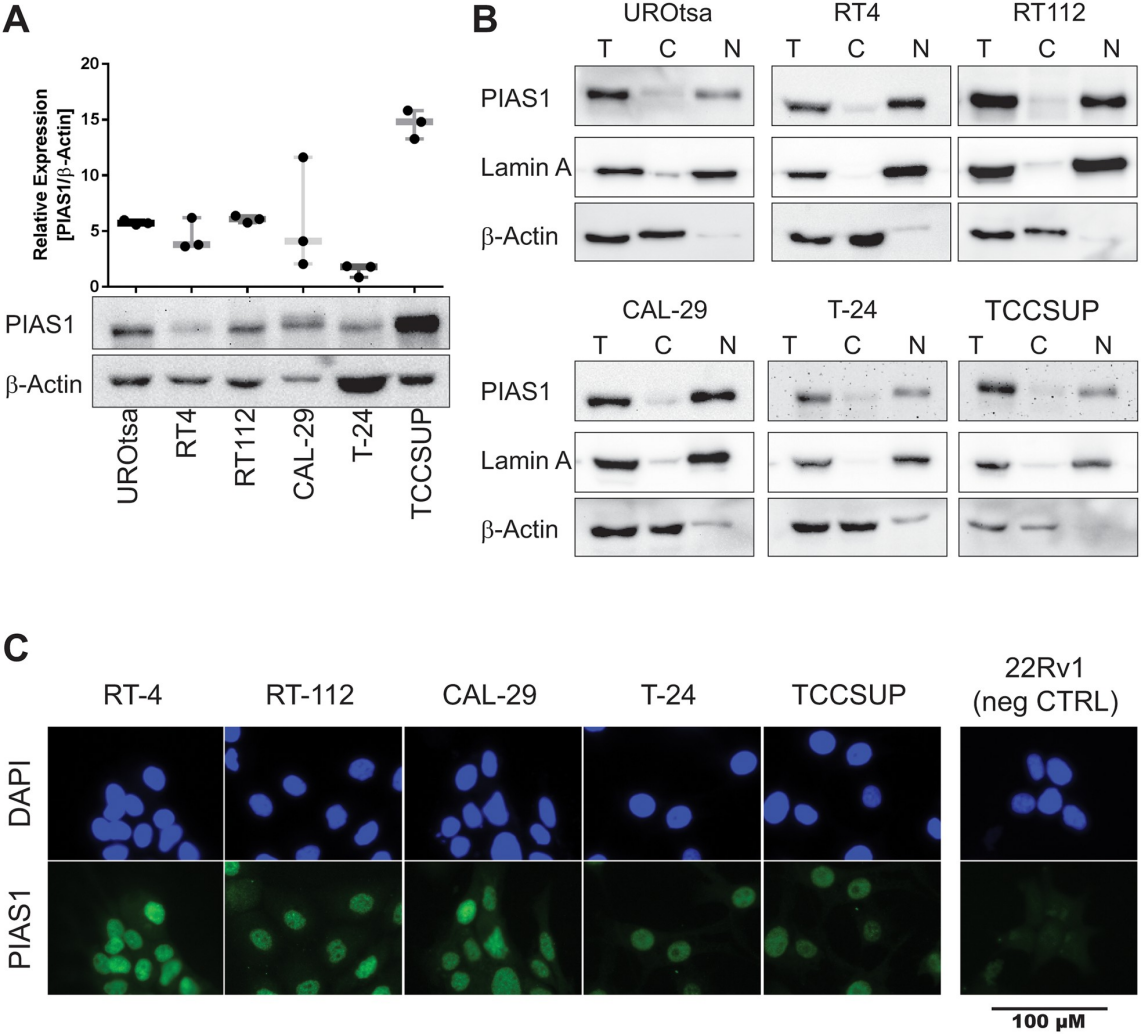
PIAS1 is expressed in benign and malignant urothelial cell lines and shows nuclear localization. (A) Western blot analysis of PIAS1 protein levels of different bladder cancer cell lines (n = 3). PIAS1 levels were normalized on β-Actin. Data are expressed as mean±SD of three independent experiments. (B) Localization PIAS1 was determined by cytoplasmatic and nuclear fractionation in several urothelial cell lines. β-Actin was used as cytoplasmic and lamin A was used as nuclear marker. (C) PIAS1 nuclear localisation (green) in the urothelial cell lines was confirmed by immunofluorescence. Counterstaining of the nuclei was performed with DAPI staining (blue).

PIAS1 protein expression in urothelial cell lines was determined by western blot. Antibody specificity (S1A Fig) was determined by using the PCa cell lines 22Rv1 (PIAS1 negative) and PC3 (PIAS1 positive) [20]. As shown in Fig 2A PIAS1 protein expression could be detected in all benign and malignant cell lines. Nevertheless, PIAS1 protein expression was heterogeneous. TCCSUP showed the highest expression, whereas T-24 showed the lowest expression. Furthermore, cell fractionation (Fig 2B) and immunofluorescence (Fig 2C) methods were applied to determine the subcellular localization of PIAS1. Both methods confirmed PIAS1 expression predominantly in the nuclei of all tested cell lines (Fig 2B and 2C).

### PIAS1 does not affect cell viability or colony formation ability in urothelial cell lines

**Fig 3:**
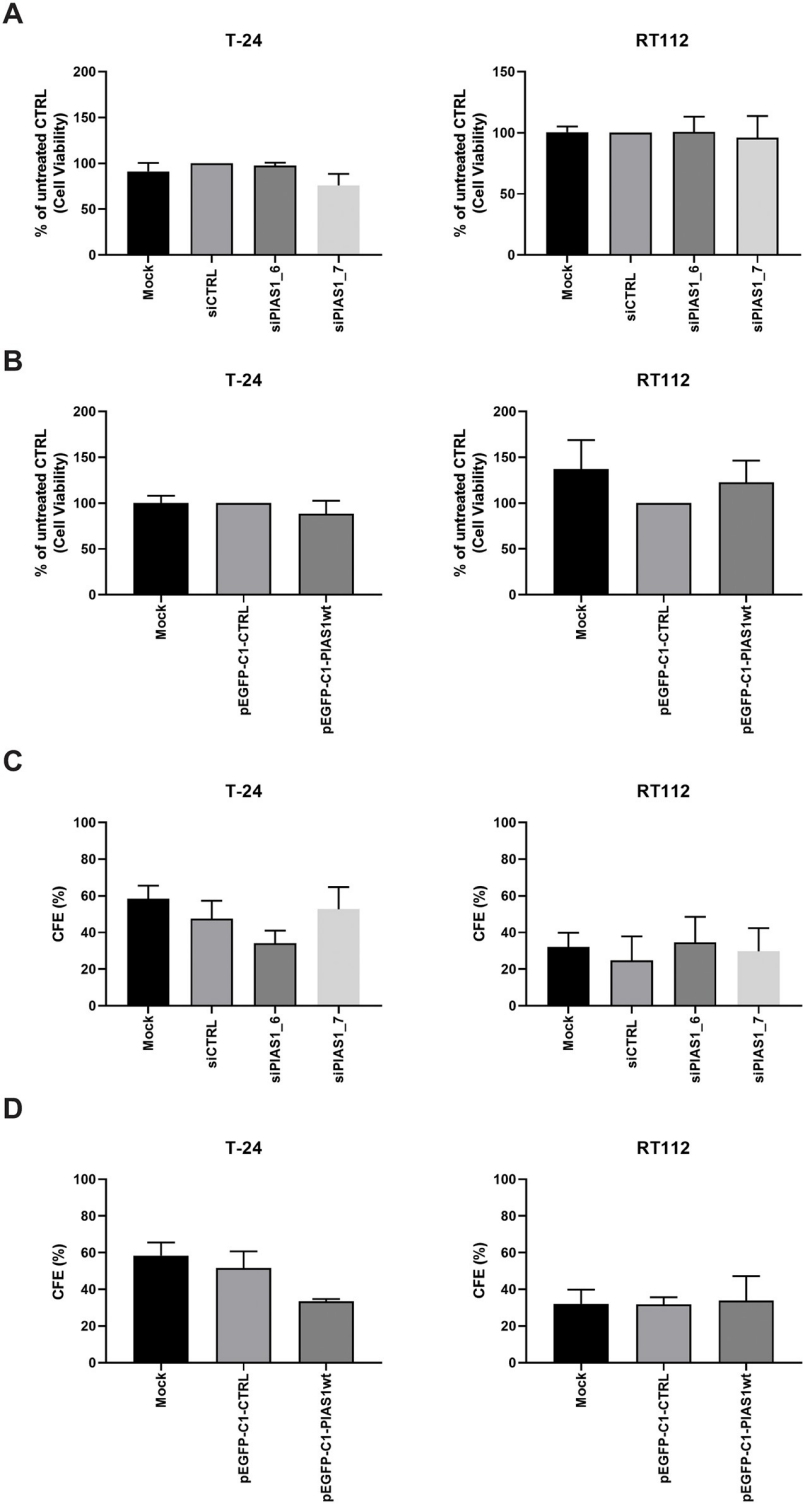
PIAS1 down-regulation or overexpression does not change cell viability or clonogenic potential of T-24 and RT112. (A) Cell viability after PIAS1 down-regulation in T-24 and RT112 was measured by using the alamar blue assay. Data are expressed as fold changes of siCTRL and are the mean±SEM of three independent experiments. (B) Cell viability after PIAS1 overexpression in T-24 and RT112 was measured by using the alamar blue assay. Data are expressed as fold changes of siCTRL and are the mean±SEM of three independent experiments. (C) Effect of PIAS1 down-regulation on colony formation ability of T24 and RT112 cells was assessed by counting the number of colonies after 10 days. Data are expressed as mean±SEM of three independent experiments (D) Effect of PIAS1 overexpression on colony formation ability of T24 and RT112 cells was assessed by counting the number of colonies after 10 d. Data are expressed as mean±SEM of three independent experiments.

For the following experiments RT112 with an average PIAS1 expression and T24 with a low PIAS1 expression were chosen. PIAS1 is reported to play a crucial role in the proliferation of PCa cells, possibly through regulation of NFκB and STAT1, which are known regulators of cellular proliferation and apoptosis in several tumour models [10, 20–22]. To investigate the role of PIAS1 in cell viability in human urothelial cancer cell lines four siRNAs were tested. All siRNAs decreased PIAS1 mRNA expression (S1C Fig) and subsequently protein expression in RT112 cells (S1D Fig). For subsequent biological experiments siPIAS1_6 and siPIAS1_7 were chosen. In addition, overexpression of PIAS1 using the pEGFP-C1-PIAS1wt could be shown in T24 and RT112 (S1E Fig). In contrast to the findings in PCa, knock down or overexpression of PIAS1 did not influence the viability of urothelial cancer cell lines (Fig 3A and 3B). In line with this result knockdown or overexpression of PIAS1 in RT112 and T24 cells did not result in a change in number of colonies formed after 10 d in a clonogenic assay (Fig 3C and 3D).

### PIAS1 knock down does not affect cell migration in urothelial cell lines

**Fig 4:**
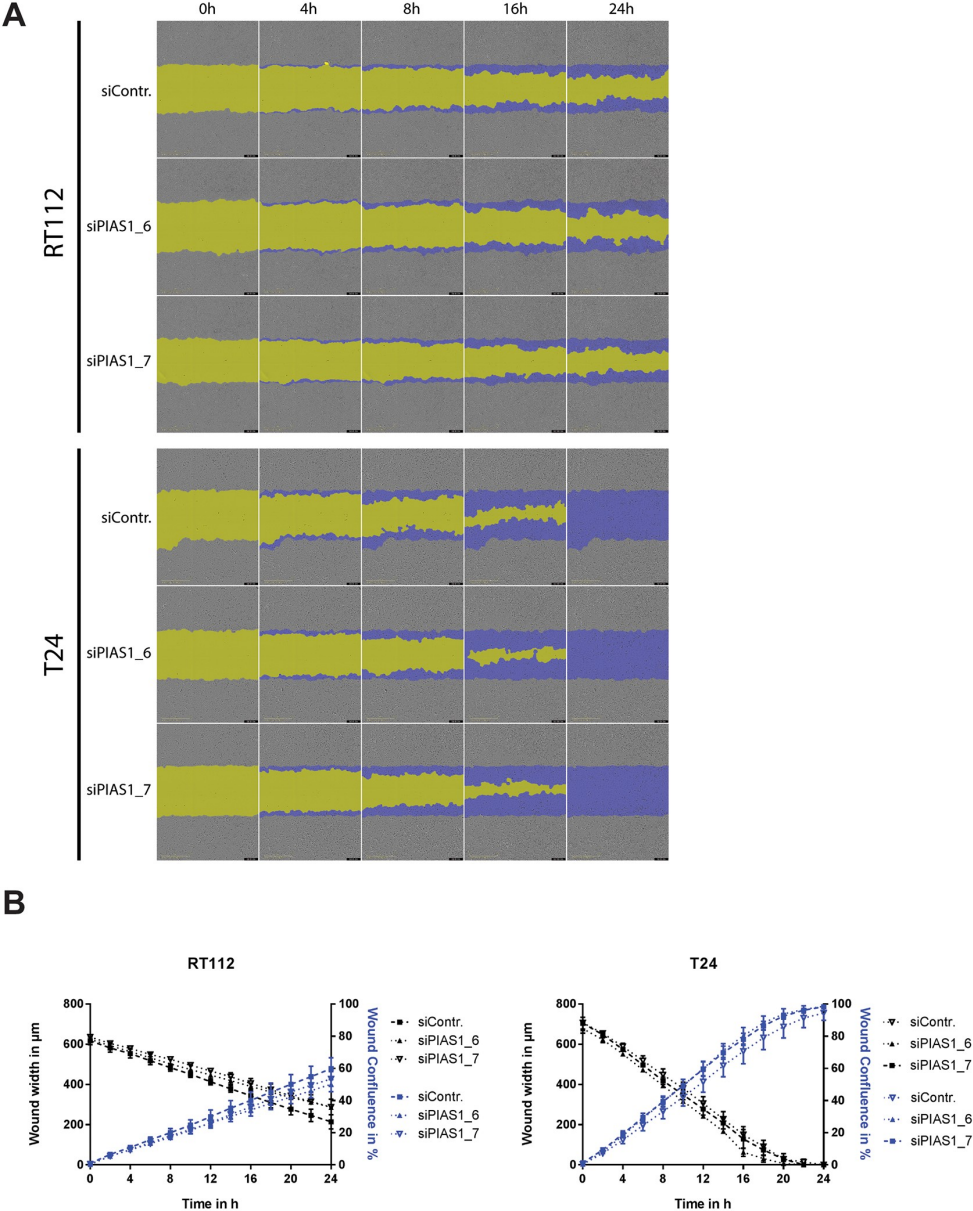
Downregulation of PIAS1 does not affect cell migration in RT112 and T24 cells. Wound heal assays after PIAS1 knock down. (A) Representative pictures of wound closure at 0, 4, 8, 12, and 24 h after scratching. (B) Statistical analysis of the wound heal assays in T24 cells and RT112 cells after siRNA transfection. Data are expressed as wound width in μm and wound confluence in % and are the mean±SEM of three independent experiments.

Migration plays an important role in metastasis [23]. To assess the role of PIAS1 in cell migration wound heal assays after knock down were performed. As shown in Fig 4 no change in migration could be observed after PIAS1 knockdown compared to the control siRNA in RT112 and T24 (Fig 4).

### PIAS1 does not influence cell survival after cisplatin treatment

**Fig 5:**
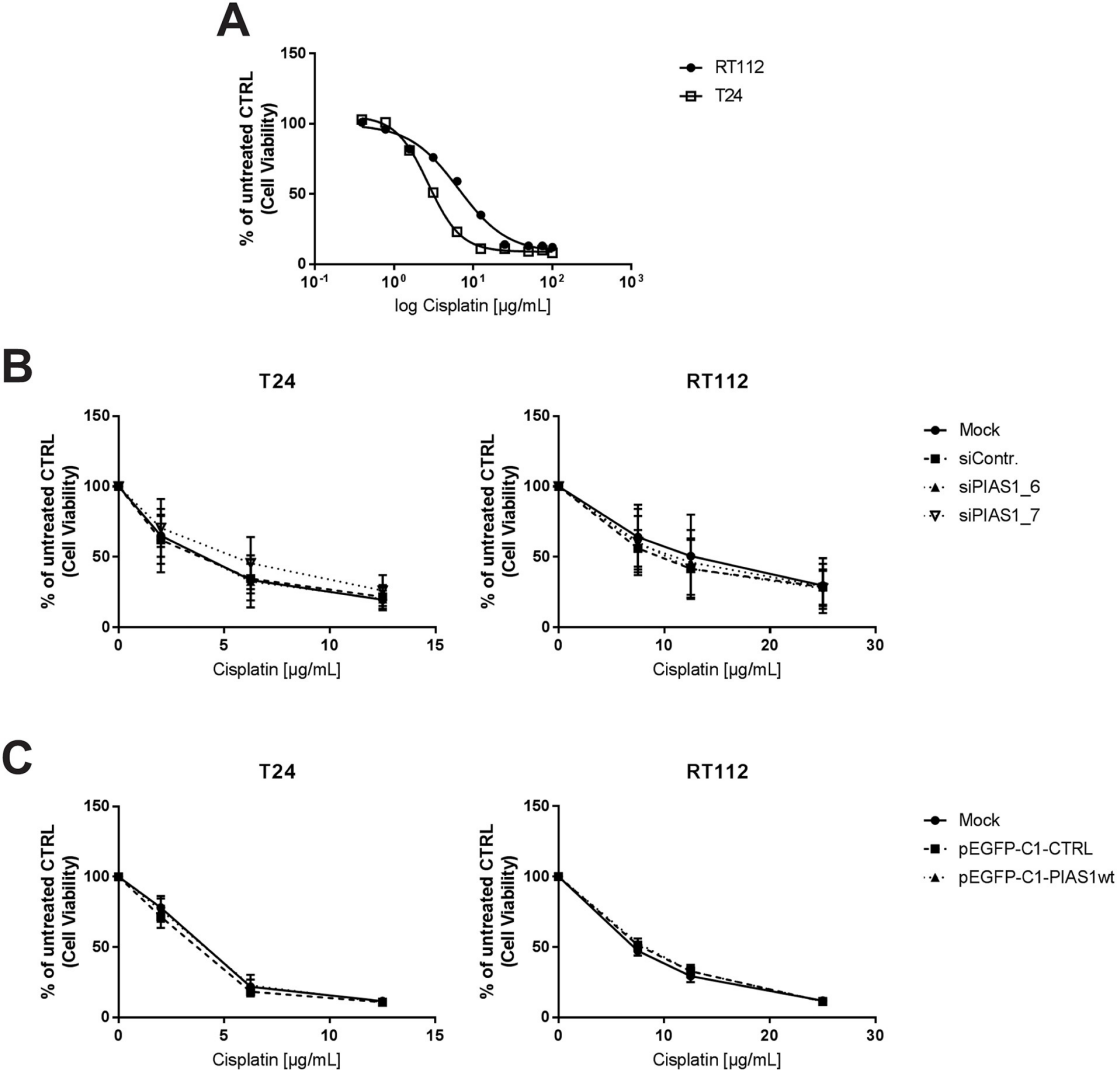
PIAS1 does not influence cell survival after cisplatin treatment. (A) IC50 values of T24 (2,71 μg/ml) and RT112 (6,5 μg/ml) were determined by treating the cells with several concentration of Cisplatin (0,5–100 μg/mL) and measuring cell viability using the alamar blue assay. Nonlinear Regression analysis was performed using GraphPad Prism. (B) Dose response curves for cell viability of T24 and RT112 after simultaneous depletion of PIAS1 and treatment with several concentrations of Cisplatin was measured by using the alamar blue assay. Data are expressed as fold changes of untreated CTRL and are the mean±SEM of three independent experiments. (C) Dose response curves for cell viability of T24 and RT112 after simultaneous overexpression of PIAS1 and treatment with several concentrations of Cisplatin was measured by using the alamar blue assay. Data are expressed as fold changes of untreated CTRL and are the mean±SEM of three independent experiments.

Cisplatin causes the development of DNA cross-links, e.g. 1,2-intrastrand cross-links of purine bases which result in apoptotic cell death [24]. Several studies have reported that PIAS1 may be involved in DNA cross-link repair [7, 25]. To explore if PIAS1 expression influences the effects of Cisplatin on cell viability, RT112 and T24 cell lines with overexpression or siRNA knock down of PIAS1 were treated with Cisplatin. To assess a range for Cisplatin treatment an IC50 for Cisplatin was determined in RT112 and T24 (Fig 5A). RT112 showed an IC50 of 6.5 μg/ml and T24 of 2.71 μg/ml. Therefore, a range for RT112 from 0–25 μg/ml was chosen and for T24 a range from 0–12 μg/ml. However, cell viability assays showed no change in the dose response experiments in cisplatin-treated RT112 and T24 cell lines after PIAS1 knock down (Fig 5B) or overexpression (Fig 5C) compared to controls.

### PIAS1 does not influence cell survival and clonogenic recovery after irradiation

**Fig 6:**
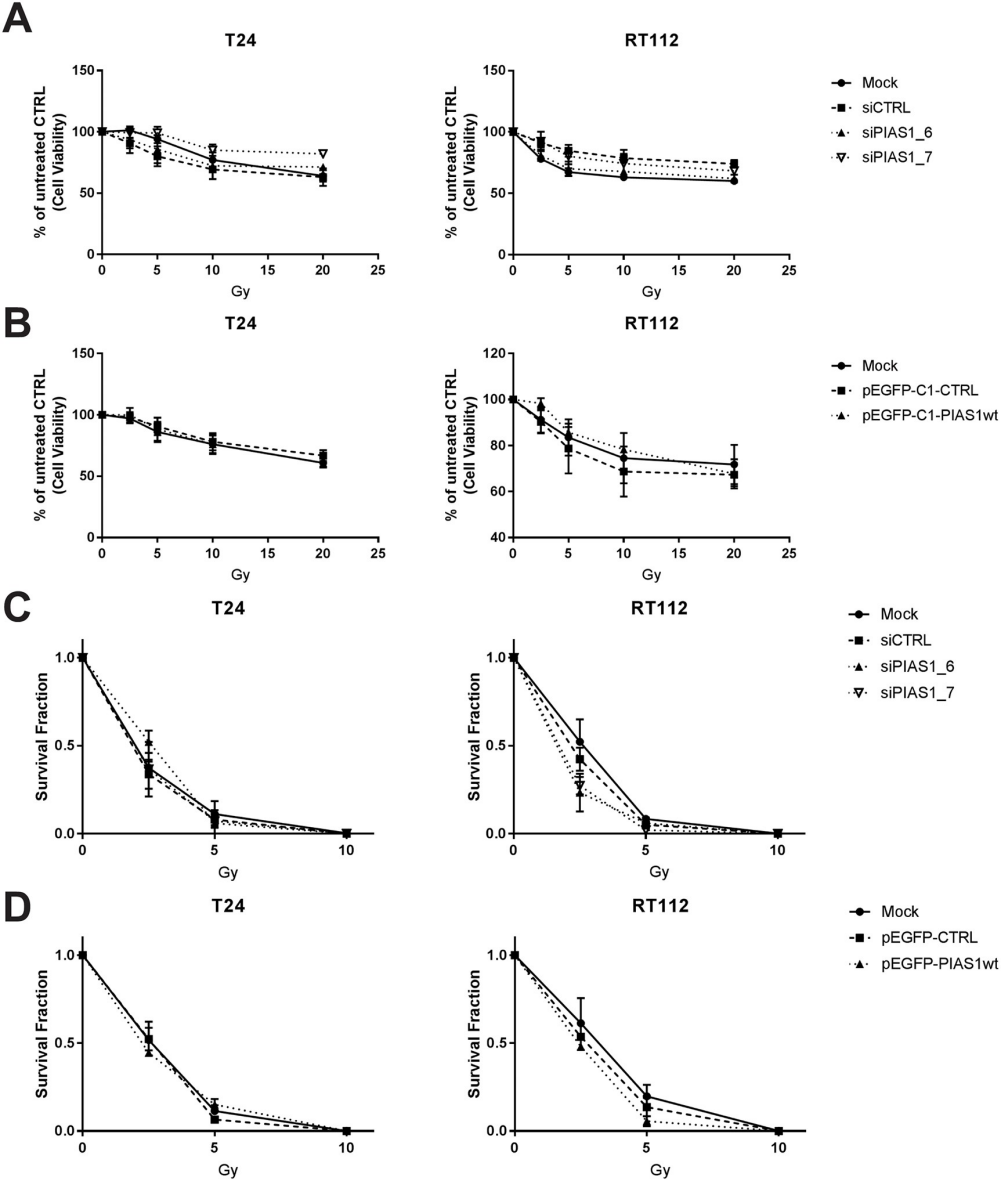
PIAS1 does not influence cell survival and clonogenic recovery after irradiation. (A) Dose response curves for cell viability of T24 and RT112 after simultaneous depletion of PIAS1 and irradiation (0, 2.5, 5, 10, 20 Gy) was measured by using the alamar blue assay. Data are expressed as mean±SEM of three independent experiments. (B) Dose response curves for cell viability of T24 and RT112 after simultaneous overexpression of PIAS1 and irradiation (0, 2.5, 5, 10, 20 Gy) was measured by using the alamar blue assay. Data are expressed as fold changes of untreated CTRL and are the mean±SEM of three independent experiments. (C) Clonogenic revovery assays were performed with T24 and RT112 after simultaneous depletion of PIAS1 and irradiation (0, 2.5, 5, 10, 20 Gy) and survival fractions are expressed as mean±SEM of three independent experiments. (D) Clonogenic recovery assays were performed with T24 and RT112 after simultaneous overexpression of PIAS1 and irradiation (0, 2.5, 5, 10, 20 Gy) and survival fractions are expressed as fold changes of untreated CTRL and are the mean±SEM of three independent experiments.

A complex network of proteins must assemble to initiate the Process of DNA damage repair (DDR) successfully. Such proteins include the RAD52 epistasis group of proteins which have been shown to be regulated by PIAS1 [26, 27]. However, it is currently unknown whether PIAS1 influences double-strand break DNA repair in urothelial cells. Therefore, RT112 and T24 cell lines with knocked down or overexpressed PIAS1 were exposed to several doses of gamma irradiation and cell viability was assessed. Neither knock down (Fig 6A) nor overexpression (Fig 6B) of PIAS1 showed any significant change in cell viability in the tested cell lines after irradiation with 2.5, 5, 10, or 20 Gy compared to the control siRNA. Clonogenic assays are commonly used to investigate survival of irradiated cancer cells, whereas cell viability assays are usually used to analyse chemosensitivity or toxicity of drugs in human tumour cell lines [28]. Therefore, clonogenic recovery assays were performed with irradiated RT112 and T24 cell lines with overexpressed or knocked down PIAS1. However, the clonogenic recovery assays showed no difference with knock down (Fig 6C) or overexpression (Fig 6D) of PIAS1 compared to the controls.

## Discussion

In the recent years studies have revealed an important role of SUMOylation in tumourigenesis, tumour progression, and metastasis [29]. Especially TGF-β signalling seem to be highly regulated by SUMOylation in UC [30]. Tan and collegues revealed that SUMOylation of SnoN by the SUMO E3 ligase inhibits TGF-β induced epithelial-mesenchymal transition (EMT) and invasion [31]. In addition, the deSUMOylation protease SENP2 has shown to be involved in metastiatic progression by regulating EMT and MMP13 expression in UC [32, 33]. The multifunctional E3 SUMO-protein ligase PIAS1 has been reported to play a key role in the regulation of many cellular pathways [8]. In addition to its regulatory role of STAT1 and NFκB, there is evidence that it also regulates the expression and transcriptional activity of other transcription factors such as AR [34, 35]. PIAS1 has also been shown to be involved in several important cellular processes including cell cycle control and DNA damage repair (DDR) and therefore may play an important role in genomic stability and tumourigenesis [4, 25, 27, 36]. Due to these findings it is not surprising that several groups demonstrated that PIAS1 is dysregulated and involved in tumour survival. In breast cancer, PIAS1 regulates tumourigenesis through gene silencing and may serve as a potential survival biomarker [11, 37]. In colon cancer PIAS1 has been shown to repress the cancer stem cell population. Therefore, reduced expression of PIAS1 is associated with colon cancer development [38, 39]. Several studies from Puhr et al. and Höfer et al. demonstrated a crucial role in PCa survival, AR regulation and as determinant of poor survival [9, 10, 20].

In this study, the role of PIAS1 UC was investigated for the first time. Given that sumoylation of proteins plays a critical role in the regulation of protein activity, and the TCGA database showed that PIAS1 is WT in urothelium, we hypothesized that modulation of PIAS1 might result in biological responses [25, 40]. Analysis of several public available arrays revealed that PIAS1 mRNA expression is not changed between begin and malignant tumour areas. Also, there was no difference between muscle invasive and non-muscle invasive samples. These results show that in contrast to colon cancer, breast cancer and PCa [9, 37, 39], PIAS1 might not be a potential marker for UC. Furthermore, screening of the commonly used cell lines revealed no significant differences in the expression of the PIAS1 protein in UC. However, it has been important to validate protein expression levels using primary patient specimens.

Whilst not completely clarified, data in PCa models show that upregulation of p21 by PIAS1 knock down leads to cell cycle arrest [20]. It is hypothesized that PIAS1 regulates p21 via p73 in PCa cells as it is reported to be involved in cell cycle regulation by SUMOylation of the tumour suppressors p53 or p73 [20, 41, 42]. This regulation was proven by Munarriz et al., who demonstrated that PIAS1 is a check point regulator during the S phase of the cell cycle by SUMOylating p73 [42]. In contrast to these findings, knock down of PIAS1 in the tested UC cell lines showed no effect on viability or clonogenic potential. These data could be explained by the apparent lack of p73 in bladder cancer tissue and cell lines as p73 mediated cell cycle control is not present in these cells [42–44]. Due to these findings we hypothesize that PIAS1 is not involved in p21, p53 and p73 regulation in UC. However, further investigations are necessary to confirm this finding.

Cisplatin is a major frontline drug in the treatment of lung, colorectal, ovarian, head-and-neck, and UC and has been used in the clinic since 1978 [45]. It kills cancer cells by creating DNA cross-links which block cell division and result in apoptotic cell death [24]. The cisplatin-induced damage is repaired by nucleotide excision repair which has also been shown to be regulated by PIAS1 [46]. However, knock down and overexpression of PIAS1 did not influence the sensitivity to cisplatin in UC and therefore seems not to be involved in resistance to the drug.

Also, other DNA damage repair (DDR) mechanisms have been demonstrated to be regulated by PIAS1 [26, 27]. To see if there is a general impact of PIAS1 on DNA damage repair, UC cells with overexpressed and down regulated PIAS1 were exposed to several doses of gamma irradiation. Our results demonstrate that PIAS1 is also not involved in DNA repair mechanisms triggered by gamma irradiation as it has been shown in breast cancer [6]. A possible explanation may be the high levels of mutations and alterations in the DNA repair pathways and cell cycle control genes in bladder cancer [47]. These mutations may alter the normal response to DNA damage as induced here by cisplatin and irradiation and therefore not crosstalk with PIAS1.

In the present study, we have demonstrated that PIAS1 plays a minor role in cell survival and DNA repair within urothelial cancer cells *in vitro*. This result was surprising when compared with the results of other studies from different tumour types, and could demonstrate that PIAS1 functions differently or that PIAS1 mediated DDR is non-functional in urothelial cancers. Due to the complex nature of the PIAS1 network it is difficult to explain the lack of effects shown here, however, there is evidence that members of the PIAS family such as PIAS4 or other E3 SUMO ligases may be able to compensate the role of PIAS1 [6, 48]. There is also the possibility that interaction PIAS1 partners such as STAT1 or NFκB are not such critical survival factors in UC compared to other entities. Summed-up, the *in vitro* data presented here do not support the role of PIAS1 as a therapeutic target in UC cancer or as biomarker as previously shown in different entities [9, 10, 37].

## Supporting information

S1 Fig Establishment of PIAS1 knock down and overexpression. (A) Testing of the antibody specify with PIAS1 negative 22Rv1 cells and PIAS1 positive PC3 cells. (B) Visual proof of transfection efficiency in RT112 (C) Establishment of different siRNAs against PIAS1 on mRNA (D) Proof of siRNA knock down efficiency on protein level after 24 h, 48 h, and 72 h in T24 cells and RT112 cells (E) Establishment of PIAS1 overexpression in T24 cells and RT112 cells.
(TIF)
