# Supplementary figures and images for: PIAS1 is not suitable as a urothelial carcinoma biomarker protein and pharmacological target
Source: PLoS One. 2019 Oct 22;14(10):e0224085. doi: 10.1371/journal.pone.0224085 (PMC6804980; doi:10.1371/journal.pone.0224085)

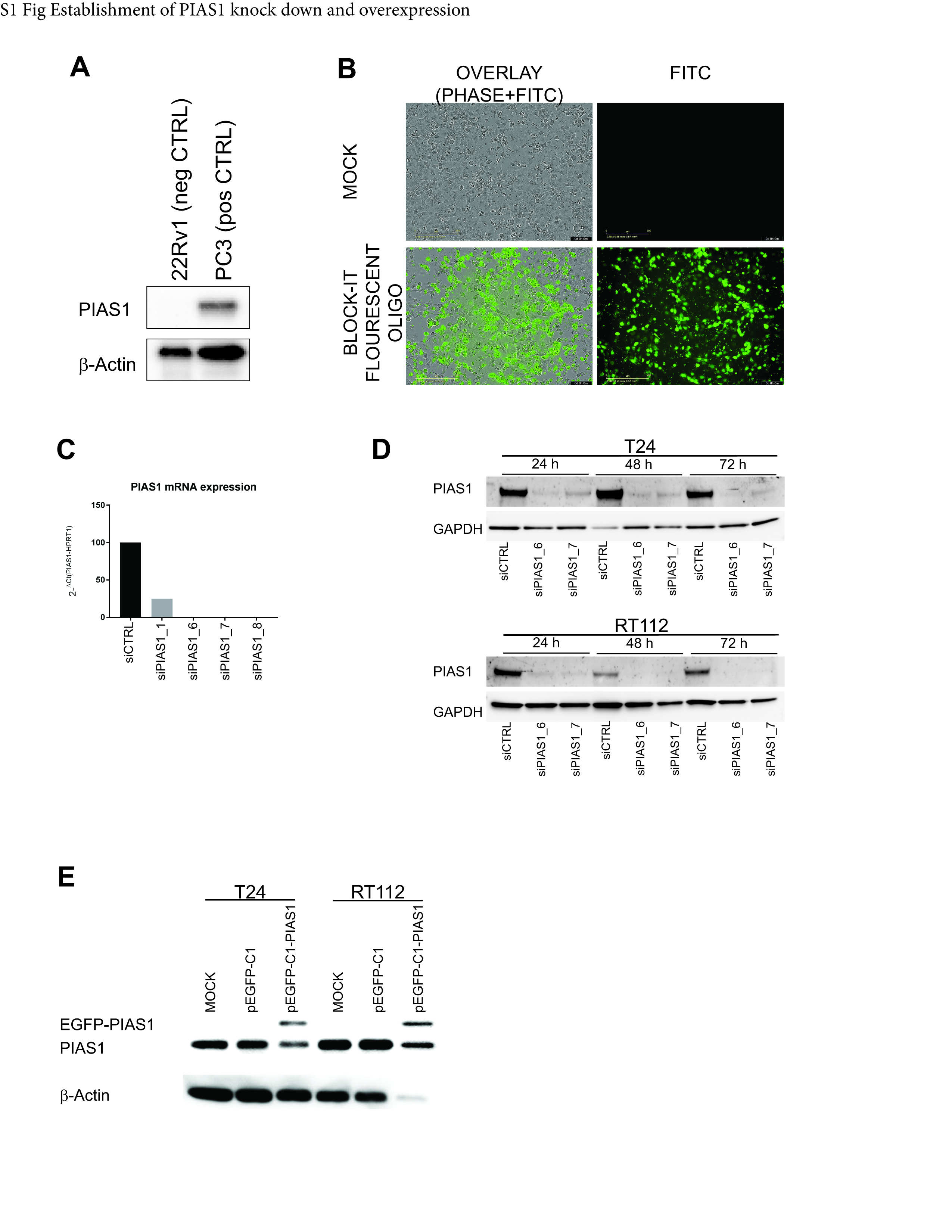

Supplement: S1 Fig — (A) Testing of the antibody specify with PIAS1 negative 22Rv1 cells and PIAS1 positive PC3 cells. (B) Visual proof of transfection efficiency in RT112 (C) Establishment of different siRNAs against PIAS1 on mRNA (D) Proof of siRNA knock down efficiency on protein level after 24 h, 48 h, and 72 h in T24 cells and RT112 cells (E) Establishment of PIAS1 overexpression in T24 cells and RT112 cells. (TIF) [file pone.0224085.s001.tif]
